# Supplementary material for: CIViC MCP: integrating large language models with the Clinical Interpretations of Variants in Cancer
Source: Bioinform Adv. 2026 Jul 29;6(1):vbag209. doi: 10.1093/bioadv/vbag209 (PMC13466716; doi:10.1093/bioadv/vbag209)
Supplement: Supplementary file 1 [file vbag209_supplementary_data.docx]

**Supplementary Data**

**Supplementary Figure 1.** Install the custom connector to interface with the CIViC MCP server. Claude will now have access to the MCP server during chats.

**Supplementary Figure 2.** Each panel corresponds to one retrieval condition: GPT-5 alone (left), GPT-5 + MCP (center), and GPT-5 Agent Mode (right). For every triplet, the model classified each of the evidence type-significance options as "Supports," "Does not Support," "Contradicting," or "No Evidence." Rows give the true CIViC label and columns give the model's prediction. Cells on the diagonal (green) are correct classifications and off-diagonal cells (red) are misclassifications, with color intensity scaling with the number of decisions in each cell.

**Prompt 1.**

**System Prompt GPT.**

You are an expert biomedical annotator trained to assess variant evidence from the Clinical Interpretations of Variants in Cancer (CIViC) knowledgebase given a specific gene variant + cancer + therapy.

**System Prompt GPT + MCP.**

You are an expert biomedical annotator trained to assess variant evidence from the Clinical Interpretations of Variants in Cancer (CIViC) knowledgebase given a specific gene variant + cancer + therapy. Use the tools to answer oncology questions for the CIViC knowledgebase.

**User Prompt**

User: For this exact combination of gene variant: {variant}, cancer type: {cancer}, therapy: {therapy}, determine whether CIViC contains matching records for each evidence type + significance below, and whether those records support that significance.

Answer choices:

A. Supports

B. Does Not Support

C. No Evidence

CONTEXT-SENSITIVE EVIDENCE RULES (important):

- Treat the user's inputs (molecular profile, disease, therapy) as REQUIRED filters.

- Only count CIViC records that explicitly match ALL user-specified fields:

- Disease must match exactly.

- Therapy must match exactly as a set (order does not matter).

- Exception: if Therapy Interaction Type is SUBSTITUTES, overlap is sufficient only for single-drug queries; for multi-drug queries the full combination must match.

- Molecular Profile matching:

- If the QUERY MP contains "AND": only include evidence MPs that contain ALL components (no partial matches).

- If the QUERY MP contains "OR": include evidence MPs if ANY component matches.

- Otherwise (single MP): include evidence MPs that match that term; do NOT include evidence MPs with "AND".

TASK:

- For each item (<evidence type> — <significance>):

- Return A if there exists ≥1 matching record with evidenceDirection = SUPPORTS.

- Return B if there exists ≥1 matching record with evidenceDirection = DOES_NOT_SUPPORT.

- Return A,B if both SUPPORTS and DOES_NOT_SUPPORT exist for that item.

- If any matching record has evidenceDirection = NA / N/A / missing, treat direction as unknown and return A,B for that item.

- Return C only if there are zero matching records for that evidence type + significance.

- The significance label "N/A" refers ONLY to CIViC records whose significance field is NA or N/A (not UNCERTAIN_SIGNIFICANCE).

Output exactly one line per item below (23 lines), in this exact format:

<evidence type> — <significance>: <A|B|A,B|C>

Items (use exactly this order, and exactly these labels):

predictive — sensitivity_response: <A|B|A,B|C>

predictive — resistance: <A|B|A,B|C>

predictive — adverse_response: <A|B|A,B|C>

predictive — reduced_sensitivity: <A|B|A,B|C>

predictive — N/A: <A|B|A,B|C>

prognostic — better_outcome: <A|B|A,B|C>

prognostic — poor_outcome: <A|B|A,B|C>

prognostic — N/A: <A|B|A,B|C>

diagnostic — positive: <A|B|A,B|C>

diagnostic — negative: <A|B|A,B|C>

predisposing — predisposition: <A|B|A,B|C>

predisposing — protectiveness: <A|B|A,B|C>

predisposing — uncertain_significance: <A|B|A,B|C>

predisposing — N/A: <A|B|A,B|C>

oncogenic — oncogenicity: <A|B|A,B|C>

oncogenic — protectiveness: <A|B|A,B|C>

oncogenic — N/A: <A|B|A,B|C>

functional — gain_of_function: <A|B|A,B|C>

functional — loss_of_function: <A|B|A,B|C>

functional — unaltered_function: <A|B|A,B|C>

functional — neomorphic: <A|B|A,B|C>

functional — dominant_negative: <A|B|A,B|C>

functional — unknown: <A|B|A,B|C>

FINAL OUTPUT RULES:

- Output ONLY the 23 lines.

- No prose, no markdown, no explanations.

**Methods:**

**GPT-5 MCP Implementation Details**

GPT-5 was accessed through the API with the model version gpt-5-2025-08-07, corresponding to the first available release. GPT-5 was run with default parameters. Unlike most alternative models, GPT-5 does not expose a temperature parameter, limiting strict reproducibility. Agent orchestration used the OpenAI Agents SDK for Python with the OpenAI client. MCP tools were defined with FastMCP and launched as a subprocess via the fastmcp CLI over the stdio transport, under Python 3.11.7. Tool use was not enforced: ModelSettings did not override the SDK's default tool_choice="auto", so the model decided on each turn whether to invoke a tool, and Runner.run() was called without an explicit max_turns, leaving the SDK's default cap of 10 model-call/tool-execution cycles per query. Each query instantiated a fresh MCP server subprocess and a fresh agent run, with no tool-call history shared across items.

**Agent Mode Evaluation**

Unlike the API, ChatGPT Agent mode does not expose a selectable or pinnable model version. The underlying model is whichever agentic model OpenAI had deployed to the ChatGPT web interface during the access window. We therefore specify Agent mode by its access method and dates rather than a version string: it was accessed through the ChatGPT web UI from March 2 to 7, 2026. Because Agent Mode responses were substantially slower and subject to monthly usage limits, the benchmark was conducted across four paid ChatGPT accounts using a standardized protocol. To reduce user-specific bias, personalization features, including memory, were disabled for all accounts before testing. All evaluation prompts were conducted within a single project, with each prompt submitted in a fresh conversation window to minimize carryover from prior chat context. Response time was measured using the ChatGPT Timestamp Chrome extension, because the ChatGPT interface does not natively display message timestamps. Timing was defined as the interval from prompt submission to completion of the final Agent Mode response. To increase throughput, some prompts were run concurrently in separate tabs on the same account. This may have slightly inflated measured response times relative to strictly sequential single-session use. This consideration affects the timing comparison but not the classification outputs themselves.

**Data Availability**

An archived snapshot of the evaluation repository is permanently available on Zenodo (DOI: 10.5281/zenodo.20938836), with the actively maintained version hosted on GitHub at <https://github.com/griffithlab/civic-mcp-server>. The GitHub README explains where each data file is located to make them more readily available. All relevant evaluation files are located under eval_QA_experiment/data. The file agent_mode_run_results.csv contains the agent mode prompts and responses; the associated chat URLs were created but may no longer be available. QA_triplet_dataset.csv contains the 100 unique (Molecular Profile, Disease, Therapy) triplets along with their expected answers.

All CIViC queries were issued against the live public APIs in March 2026. Because CIViC is an actively curated resource, retrieved annotations, and therefore exact metric values, may differ when queries are repeated at a later date.

**Table 1. GPT-5 + MCP Significance Results (Excluding “No Evidence”)**

| **Evidence Type** | **Significance** | **N** | **Precision** | **Recall** | **F1** |
| --- | --- | --- | --- | --- | --- |
| Predictive | Sensitivity | 28 | 1.00 | 0.96 | 0.98 |
| Predictive | Resistance | 17 | 1.00 | 0.94 | 0.97 |
| Predisposing | Predisposition | 13 | 1.00 | 1.00 | 1.00 |
| Oncogenic | Oncogenicity | 12 | 1.00 | 0.92 | 0.96 |
| Predisposing | N/A | 9 | 1.00 | 0.89 | 0.94 |
| Diagnostic | Positive | 8 | 1.00 | 1.00 | 1.00 |
| Predisposing | Uncertain Significance | 7 | 1.00 | 0.86 | 0.92 |
| Functional | Loss of Function | 6 | 1.00 | 1.00 | 1.00 |
| Functional | Gain of Function | 5 | 1.00 | 1.00 | 1.00 |
| Prognostic | Better Outcome | 5 | 1.00 | 1.00 | 1.00 |
| Prognostic | Poor Outcome | 5 | 1.00 | 1.00 | 1.00 |
| Functional | Dominant Negative | 4 | 1.00 | 1.00 | 1.00 |
| Predictive | Reduced Sensitivity | 3 | 1.00 | 1.00 | 1.00 |
| Diagnostic | Negative | 2 | 1.00 | 0.50 | 0.66 |
| Functional | Unaltered Function | 1 | 1.00 | 1.00 | 1.00 |

**Table 2. GPT-5 Significance Results (Excluding “No Evidence”)**

| **Evidence Type** | **Significance** | **N** | **Precision** | **Recall** | **F1** |
| --- | --- | --- | --- | --- | --- |
| Predictive | Sensitivity | 28 | 0.86 | 0.21 | 0.34 |
| Predictive | Resistance | 17 | 0.71 | 0.29 | 0.42 |
| Predisposing | Predisposition | 13 | 0.90 | 0.69 | 0.78 |
| Oncogenic | Oncogenicity | 12 | 0.25 | 0.17 | 0.2 |
| Predisposing | N/A | 9 | 0.00 | 0.00 | 0.00 |
| Diagnostic | Positive | 8 | 1.00 | 0.75 | 0.86 |
| Predisposing | Uncertain Significance | 7 | 0.00 | 0.00 | 0.00 |
| Functional | Loss of Function | 6 | 0.60 | 0.50 | 0.55 |
| Functional | Gain of Function | 5 | 0.75 | 0.60 | 0.67 |
| Prognostic | Better Outcome | 5 | 1.00 | 0.60 | 0.75 |
| Prognostic | Poor Outcome | 5 | 0.60 | 0.60 | 0.60 |
| Functional | Dominant Negative | 4 | 1.00 | 0.50 | 0.67 |
| Predictive | Reduced Sensitivity | 3 | 0.33 | 0.33 | 0.33 |
| Diagnostic | Negative | 2 | 0.00 | 0.00 | 0.00 |
| Functional | Unaltered Function | 1 | 0.00 | 0.00 | 0.00 |

**Table 3. GPT-5 Agent Mode Significance Results (Excluding “No Evidence”)**

| **Evidence Type** | **Significance** | **N** | **Precision** | **Recall** | **F1** |
| --- | --- | --- | --- | --- | --- |
| Predictive | Sensitivity | 28 | 1.00 | 0.79 | 0.88 |
| Predictive | Resistance | 17 | 1.00 | 0.88 | 0.94 |
| Predisposing | Predisposition | 13 | 0.93 | 1.00 | 0.96 |
| Oncogenic | Oncogenicity | 12 | 0.92 | 1.00 | 0.96 |
| Predisposing | N/A | 9 | 1.00 | 0.78 | 0.88 |
| Diagnostic | Positive | 8 | 0.89 | 1.00 | 0.94 |
| Predisposing | Uncertain Significance | 7 | 1.00 | 0.86 | 0.92 |
| Functional | Loss of Function | 6 | 0.86 | 1.00 | 0.92 |
| Functional | Gain of Function | 5 | 0.75 | 0.60 | 0.67 |
| Prognostic | Better Outcome | 5 | 1.00 | 1.00 | 1.00 |
| Prognostic | Poor Outcome | 5 | 1.00 | 0.80 | 0.89 |
| Functional | Dominant Negative | 4 | 1.00 | 1.00 | 1.00 |
| Predictive | Reduced Sensitivity | 3 | 1.00 | 1.00 | 1.00 |
| Diagnostic | Negative | 2 | 0.00 | 0.00 | 0.00 |
| Functional | Unaltered Function | 1 | 1.00 | 1.00 | 1.00 |
